# Methylmalonic and propionic acidemia among hospitalized pediatric patients: a nationwide report

**DOI:** 10.1186/s13023-019-1268-1

**Published:** 2019-12-16

**Authors:** Yi-Zhou Jiang, Yu Shi, Ying Shi, Lan-Xia Gan, Yuan-Yuan Kong, Zhi-Jun Zhu, Hai-Bo Wang, Li-Ying Sun

**Affiliations:** 1 National Clinical Research Centre for Digestive Diseases, Beijing Friendship Hospital, Capital Medical University, 95# Yong-an Road, Xi Cheng District, Beijing, 100050 China; 2 China Standard Medical Information Research Centre, Shenzhen, Guangdong China; 3 Clinical Trial Unit, Precision Medicine Institute, First Affiliated Hospital of Sun Yat-Sen University, Guangzhou, No.58, Zhong Shan Er Lu, Guangzhou, 510080 China

**Keywords:** Hospitalized, MMA, PA, Pediatric

## Abstract

**Background:**

Methylmalonic acidemia (MMA) and propionic acidemia (PA) are two kinds of diseases caused by inborn errors of metabolism. So far, the epidemiological data on them are limited in China. The aim of our study is to investigate the proportion and characteristics of hospitalized pediatric patients with MMA and PA in China.

**Methods:**

The data in this study were obtained from the Hospital Quality Monitoring System, a national inpatient database in China, with information on the patients hospitalized during the period from 2013 to 2017. We identified the data related to the patients who were under 18 years old and were diagnosed with MMA/PA, and extracted the information on demographic characteristics, hospital location, total cost and other related clinical presentations from the data.

**Results:**

Among all hospitalized pediatric patients with liver diseases, there were increasing trends in the proportion of individuals diagnosed with MMA or PA during the period from 2013 (0.76% for MMA; 0.13% for PA) to 2017 (1.61% for MMA; 0.32% for PA). For both MMA and PA, children under 2-year-old accounted for the highest proportion. The median of total cost per hospitalization was relatively high (RMB 7388.53 for MMA; RMB 4999.66 for PA). Moreover, most patients hospitalized in tertiary class A hospitals (MMA: 80.96%, PA: 76.21%); and a majority of pediatric patients admitted in the hospitals in Shanghai and Beijing are from outside districts. Manifestations of nervous system-related symptoms, and metabolic acidosis or anemia in laboratory findings were more common during hospitalization.

**Conclusions:**

The study is the first nationwide one in providing epidemiological and clinical information on hospitalized pediatric patients with MMA/PA. An increasing hospitalization with various presentations and a heavy financial burden were observed. In addition, geographically, the medical resources in China have been unevenly distributed.

## Introduction

Methylmalonic acidemia (MMA) (OMIM #251000, MMA mut type; OMIM #251100, MMA cblA type; OMIM #251110, MMA cblB type; OMIM #277410, MMA cblD-variant 2) and propionic acidemia (PA) (OMIM #606054), two diseases caused by inborn errors of metabolism, are the most common organic acidurias [1, 2]. The major characteristic of MMA and PA is the accumulation of toxic metabolites caused by disrupting the normal amino acid metabolism due to defects in enzyme methylmalonyl-CoA mutase (MMUT) or propionyl-CoA carboxylase (PCC) [3]. The age of onset ranges from neonatal to adult and the clinical signs of MMA and PA are usually nonspecific and diversified. Patients with complete enzyme deficiency may present symptom in the first few days of life while late-onset cases at any age (mostly before 18) [1–5]. High frequency of hospitalization occurs owing to recurrent acute episode of metabolic decompensation and multiple systemic complications involving brain, kidney and heart [1, 3, 6–8]. The most terrifying fact is that MMA and PA can result in death if they are untreated or improperly treated.

MMA and PA are both rare disorders of propionate catabolism among children. Compared with PA, MMA has been commonly reported with higher prevalence rate, but the incidence varies greatly worldwide [9–14]. However, their demographic characteristics and hospital-related information are limited. Due to the rarity of these two diseases, previous studies about the clinical presentations of them are based on case report, studies with small sample size, or literature review [1, 3, 8, 15–17].

Since China is a country with a huge population and MMA/PA are increasingly recognized diseases, the two diseases may pose a potential challenge to China’s national health system. Hence, to obtain information about the epidemiology and clinical presentations of MMA/PA is critical to the comprehensive perception of both of them and will be conducive to early diagnosis and even prognosis. Considering the low incidence and the cost of epidemiological survey, analyzing existing big data is a more rational way. Therefore, the purpose of this study was to 1) provide epidemiological information and financial burden status; 2) evaluate frequency of different clinical manifestations and laboratory results of MMA and PA hospitalizations among pediatric patients in China based on a large national database.

## Methods

### Data sources

The database we used is the Hospital Quality Monitoring System (HQMS) [18], a mandatory patient-level registration database of standardized electronic inpatient discharge records from part of tertiary and secondary hospitals in China under the administration of the Bureau of Medical Administration and Medical Service Supervision, National Health and Family Planning Commission of the People’s Republic of China, similar to Nationwide Inpatient Sample (NIS) in the United States. The automatic submittal of electronic discharge records to HQMS begun on January 1, 2013.

Physicians were in charge of completing the data on the front page of records, and the diagnosis was coded based on the International Classification of Diseases, revision 10 (ICD-10) coding system by certified professional medical coders at each participating hospital. Data quality was controlled automatically at the time of data submission.

### Data collection

The study included all hospitalizations of patients who were under 18 years of age with a primary or secondary diagnosis of underlying liver disease (including viral hepatitis, non-viral infectious liver disease, alcoholic liver disease, non-alcoholic fatty liver disease (NAFLD), liver neoplasms, autoimmune liver disease, drugs/toxin-induced liver injury, trauma, biliary atresia, metabolic liver disease, other congenital diseases, hepatic vascular/anatomical abnormalities and idiopathic portal hypertension) using ICD-10 from 1 January 2013 to 31 December 2017. Among these diseases, MMA and PA were defined with the following ICD-10 codes: MMA (E71.102), PA (E71.101), all of whose hospital discharge data were extracted and analysed retrospectively. Other diagnosis related to MMA and PA (possible symptoms/comorbidities mostly based on the proposed guidelines for the diagnosis of MMA and PA [1]) were also defined using ICD-10 and classified according to symptoms of different systems and laboratory test results.

Demographic characteristics including age, sex and residence were extracted from the front page of the hospitalization medical record. Hospital location, length of stay and expenditure were also collected.

### Statistical analysis

Continuous data were expressed as mean ± standard deviation, or as median (inter-quartile range) for highly skewed variables. Categorical variables were presented as frequency and percentage (%). Cochran-Armitage test for trend was performed to assess the trend of the proportion of MMA or PA pediatric patients.

All *P* values were 2-tailed. A *P* value less than 0.05 was considered to be significant. All analysis was performed using SAS software, version 9.4 (SAS Institute Inc., Cary, NC, United States). The maps were drawn by JMP software, version 14 (SAS Institute Inc., Cary, NC, United States).

## Results

### Proportion trend of MMA and PA from 2013 to 2017

**Fig. 1:**
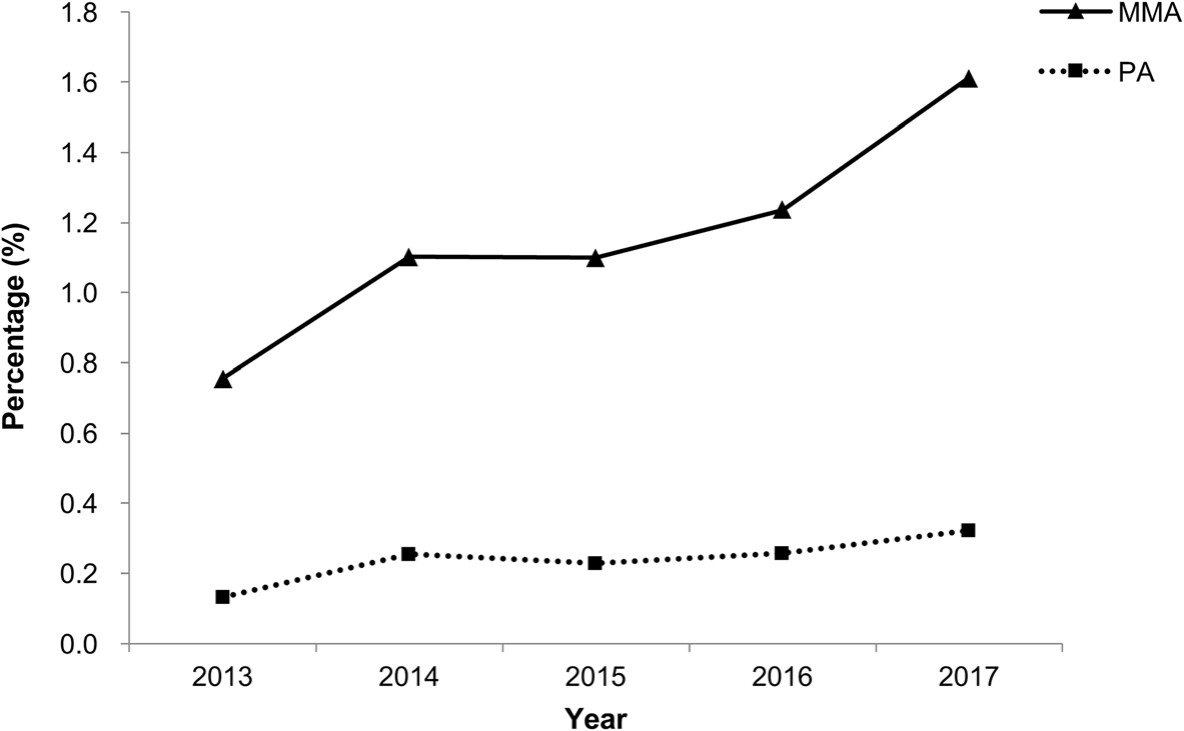
The proportion of MMA and PA in hospitalized pediatric liver disease during the period from 2013 to 2017

During the period from 2013 to 2017, total frequency of hospitalizations of the pediatric patients identified, with MMA and PA were 2610 and 538 in the database respectively. Among all hospitalized pediatric patients with liver diseases, the proportion of hospitalized pediatric patients with MMA showed an increasing trend from 2013 (*n* = 282 (0.76%)) to 2017 (*n* = 716 (1.61%)) with statistical significance (*P*_trend_ < 0.001). There was also a relatively slow upward trend in the proportion of individuals diagnosed with PA from 2013 (*n* = 49 (0.13%)) to 2017 (*n* = 143 (0.32%)) (Fig. 1). Though there was a slight decrease in 2015, the overall increasing trend was still statistically significant (*P*_trend_ < 0.001).

### Demographic characteristics of hospitalized pediatric patients

**Fig. 2:**
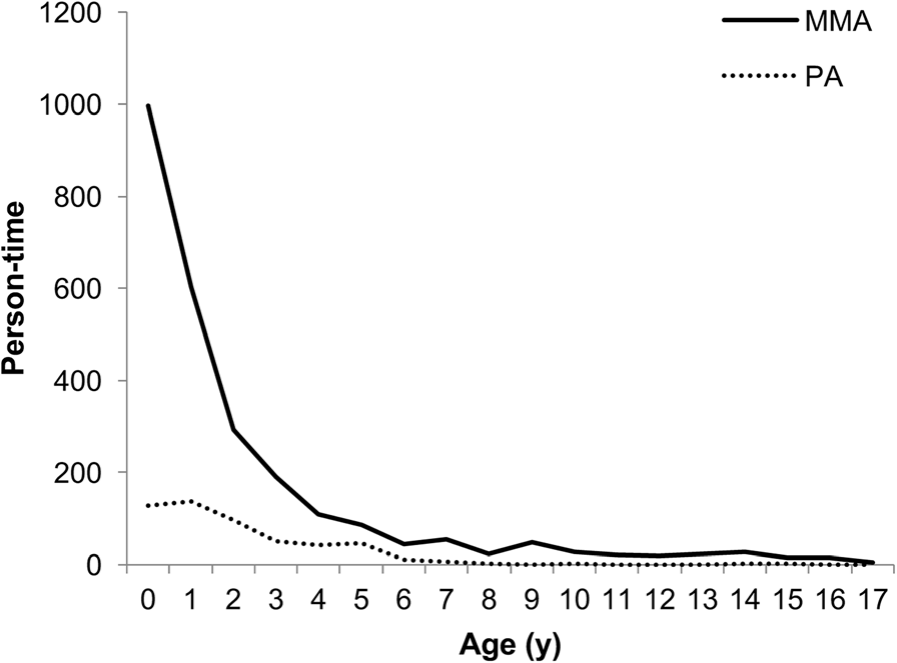
The age distribution of MMA and PA

The demographic characteristics of patients with MMA and PA were shown in Table 1. The median age (y) of patients with MMA (1.00 (0.25–3.00)) was almost equal to that of patients with PA (1.00 (0.83–3.00)). The age distribution was further analysed. The group of infants under 1-year-old had the highest proportion in the hospitalized pediatric patients with MMA, and the children under 2-year-old accounted for 61.5 percentage of all hospitalizations with MMA. Yet infants under 2-year-old accounted for 49.6% of those with PA (Fig. 2). There were more male patients than female for both MMA (Male: 57.85%, Female: 42.15%) and PA (Male: 67.66%, Female: 32.34%).

**Table 1:** Demographic characteristics of patients with MMA and PA from 2013 to 2017

	MMA	PA
Age (year)	1.00 (0.25–3.00)	1.00 (0.83–3.00)
Sex		
Male	1510 (57.85%)	364 (67.66%)
Female	1100 (42.15%)	174 (32.34%)
Length-of-stay (days)	8.0 (4.0–13.0)	7.0 (4.0–10.0)
Hospital level		
Tertiary class A hospital	2113 (80.96%)	410 (76.21%)
Tertiary class B hospital	497 (19.04%)	126 (23.42%)
Secondary hospital	0	2 (0.37%)
Type of admission		
Emergency or Referral	858 (32.87%)	161 (29.93%)
Routine	1564 (59.92%)	316 (58.74%)
Other	188 (7.20%)	61 (11.34%)
Total costs (RMB) per hospitalization	7388.53 (3298.72, 15,464.66)	4999.66 (2545.03, 10,032.57)
In-hospital death		
Yes	44 (1.69%)	12 (2.23%)
No	2566 (98.31%)	526 (97.77%)

### Hospital characteristics of delivery hospitalizations

As shown in Table 1, the median of length of stay (LOS) for MMA (8.0 (4.0–13.0)) was slightly higher than that of PA (7.0 (4.0–10.0)). Most of MMA pediatric patients (80.96%) were hospitalized in tertiary class A hospitals. There was relatively lower percentage of PA patients hospitalized in tertiary class A hospitals (76.21%). Moreover, the total cost of every hospitalization of MMA (RMB 7388.53 (IQR: 3298.72-15,464.66)) was higher than that of PA (RMB 4999.66 (IQR: 2545.03-10,032.57)). Over 50% of MMA and PA patients were admitted to hospital through routine, followed by nearly 30% through emergency or referral. The in-hospital mortality of PA was 2.23%, which was slightly higher than that of MMA (1.69%).

### Cross-district attendance in China

**Fig. 3:**
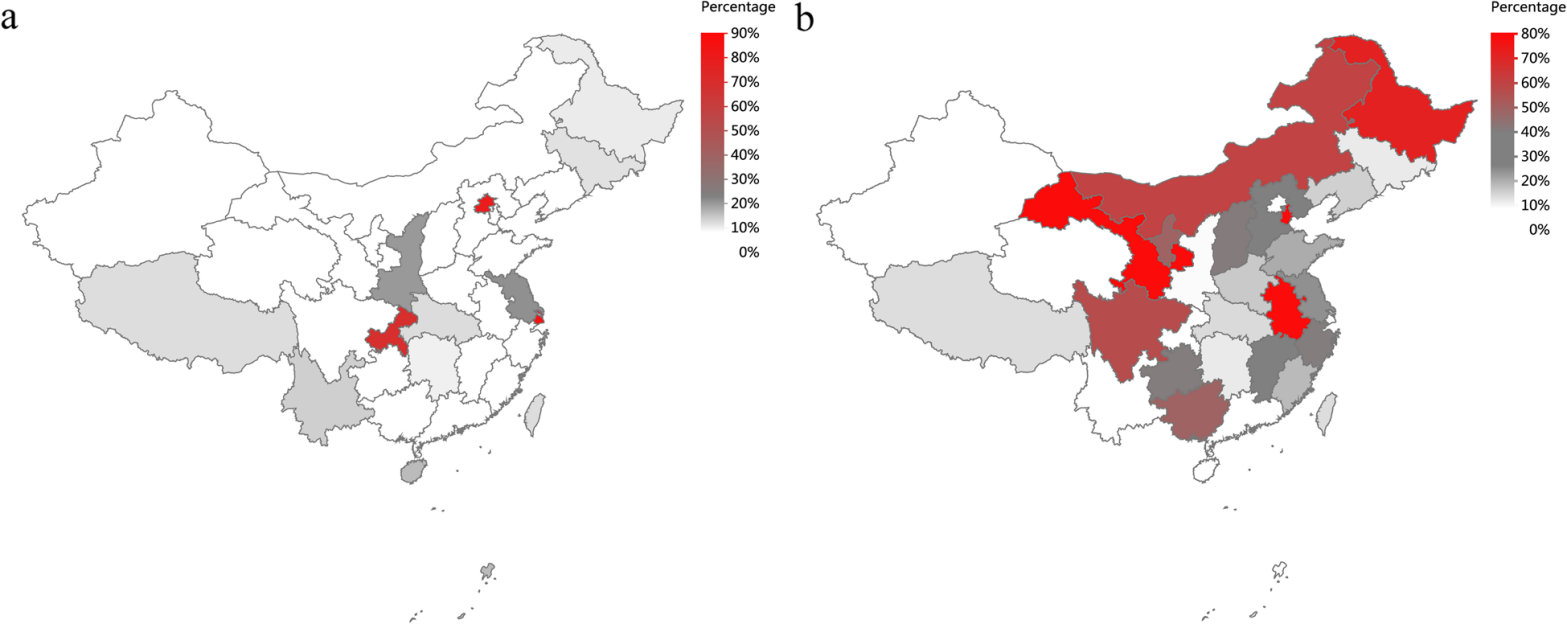
Cross-district attendance of MMA. **a**. The percentage of the hospitals in different districts which admitted MMA pediatric patients from outside districts. **b**. The percentage of MMA pediatric patients from different districts who went to the hospitals in other districts

In the relatively developed districts, there are a high proportion of patients from other districts. As shown in the Fig. 3a, 86.8% of MMA pediatric patients seen in the hospitals in Shanghai are from outside districts, followed by Beijing (80.5%), and Chongqing (70.4%). 90.0% of PA pediatric patients seen in the hospitals in Beijing are from outside districts, followed by Chongqing (86.7%), Jiangsu (83.3%) and Shanghai (60.9%).

A certain proportion of patients from some areas went to hospitals outside. Up to 82.6% of MMA pediatric patients in Gansu went to the hospitals in other districts, followed by Anhui (80.2%), Tianjin (80.0%) and Heilongjiang (72.4%) (shown in Fig. 3b). And 55.6% of PA pediatric patients in Jiangsu chose to see doctors outside, followed by Anhui (50.9%), Sichuan (42.4%) and Jiangxi (36.4%).

### Frequency of different clinical presentations and laboratory findings

The frequency of different signs and symptoms of these two diseases among all hospitalizations are listed in Table 2. Nervous system has been more susceptible for MMA/PA patients and in-patients tend to have related clinical features: seizures/epilepsy (MMA: 14.64%, PA: 14.50%), developmental delay (MMA: 7.82%, PA: 9.48%), movement disorder/dystonia (MMA: 2.84%, PA: 2.97%), and encephalopathy (MMA: 6.32%, PA: 7.43%). Patients also manifested with renal, cardiac damage; or gastrointestinal symptoms, hematologic findings, immunodeficiency; or other symptoms including hearing loss, visual deterioration and skin lesions. Compared with those with PA, patients with MMA may be more likely to have a chronic renal damage and cardiac insufficiency. It is noteworthy that prolonged QTc interval is potentially specific in PA.

**Table 2:** Frequency of different clinical presentations of MMA&PA

	MMA (*n* = 2610)	PA (*n* = 538)
Nervous system		
Seizures/ Epilepsy	382 (14.64%)	78 (14.50%)
Developmental delay	204 (7.82%)	51 (9.48%)
Encephalopathy	165 (6.32%)	40 (7.43%)
Movement disorder/Dystonia	74 (2.84%)	16 (2.97%)
Altered level of consciousness	4 (0.15%)	0
Optic atrophy	1 (0.04%)	1 (0.19%)
Kidney		
Acute renal failure	14 (0.54%)	3 (0.56%)
Chronic renal failure	20 (0.77%)	1 (0.19%)
Chronic renal insufficiency	32 (1.23%)	0
Heart		
Cardiomyopathy	100 (3.83%)	21 (3.90%)
Cardiac insufficiency	93 (3.56%)	5 (0.93%)
Arrhythmia	21 (0.80%)	8 (1.49%)
Prolonged QTc interval	0	3 (0.56%)
Gastrointestinal system		
Failure to thrive	123 (4.71%)	37 (6.88%)
Abnormal feeding behavior	66 (2.53%)	11 (2.04%)
Vomiting/Ketoacidosis	5 (0.19%)	1 (0.19%)
Pancreatitis	1 (0.04%)	0
Hepatomegaly	2 (0.08%)	0
Hematologic findings		
Neutropenia	84 (3.22%)	30 (5.58%)
Pancytopenia	8 (0.31%)	10 (1.86%)
Involvement of bone marrow	9 (0.34%)	5 (0.93%)
Immune system		
Immunodeficiency	9 (0.34%)	3 (0.56%)
Others		
Skin lesions	42 (1.61%)	8 (1.49%)
Hearing loss	27 (1.03%)	5 (0.93%)
Visual deterioration	6 (0.23%)	0

Laboratory findings are shown in Table 3. In-patients with MMA/PA, the appearance of metabolic acidosis (MMA: 13.14%, PA: 19.14%) and anemia (MMA: 19.23%, PA: 15.61%) were relatively common, followed by elevated ALT/AST/LDH, low PLT, elevated NH3, hypoglycemia, low WBC, decreased eGFR and elevated uric acid. In addition, elevated lactic acid, myocardial enzyme and EEG abnormalities were specific features in MMA.

**Table 3:** Frequency of different laboratory findings of MMA&PA

	MMA (*n* = 2610)	PA (*n* = 538)
Metabolic acidosis	343 (13.14%)	103 (19.14%)
Anemia	502 (19.23%)	84 (15.61%)
↓ PLT	66 (2.53%)	27 (5.02%)
↓ WBC	20 (0.77%)	6 (1.12%)
↑ ALT/AST/LDH	139 (5.33%)	21 (3.90%)
↑ NH3	44 (1.69%)	42 (7.81%)
Hypoglycemia	38 (1.46%)	16 (2.97%)
↓ eGFR	24 (0.92%)	5 (0.93%)
↑ Uric acid	13 (0.50%)	1 (0.19%)
↑ Lactic acid	8 (0.31%)	0
↑ Myocardial enzyme	5 (0.19%)	0
EEG abnormalities	4 (0.15%)	0

PLT platelets, WBC white blood cell, ALT alanine transaminase, AST aspartate transaminase, LDH lactate dehydrogenase, eGFR estimated glomerular filtration rate, EEG electroencephalography

## Discussion

Our study is the first nation-wide study aimed to provide epidemiological information, including the proportion, demographic feature and characteristics of hospitalized pediatric patients with MMA or PA. Although MMA and PA are rare diseases, the national database provided data on a certain amount of MMA and PA pediatric patients. Therefore, this study helps bring reliable data for our understanding of these two rare diseases.

Several of our findings are noteworthy. First, in this national dataset of hospitalized patients, the proportion of hospitalized pediatric patients with MMA or PA showed an overall increasing trend from 2013 to 2017. This phenomenon presented that there had been a growing pressure on the national health system in China. One plausible explanation is that the increase is mainly due to the accumulation of experience in recognition and diagnosis of MMA and PA over time, as MMA and PA have become the most common organic acidurias. The clinical manifestations of patients with these diseases are complex and vary in severity. They can be manifested as a single or multiple organ damages, which makes them difficult to be identified except relying on biochemical and genetic analysis to make a definite diagnosis [3, 19–21]. Case of PA was first reported in 1991 in China [22], while the biochemical diagnosis technology of MMA was not introduced from abroad until 1998 [23]. Since then, biochemistry (tandem mass spectrometry (MS/MS) and gas chromatography/mass spectrometry (GC/MS)) and genetic diagnosis has become more and more available, which has greatly decreased the chances of mistakes in diagnosis. Moreover, the screening of inborn errors of metabolism had also been more conducted for sick infants [23, 24]. And parents are actively seeking the help of doctors soon after the onset of illness in hopes of proper diagnosis and timely treatment of their kids. Another explanation is that early diagnosis seems to be associated with lower mortality rate [1], thus increases the chance of hospitalization in patients’ later life.

Though all the pediatric patients were just from the nationwide hospitalized data, the MMA-to-PA number ratio of hospitalized admission might represent overall incidence ratio of MMA and PA in the population of China to some extent. In the worldwide context, the actual incidence of MMA and PA is still unknown in some areas [11]. The estimated live-birth incidence of MMA is 1:70,000, nearly 1.5 times as that of PA (1:105,000) in the US [25]. In Italy, the ratio increases to 2.7 (MMA with 1:61,700; PA with 1:166,000) [9]. However, our study showed that the admissions of patients with MMA were 5 times as many as those of PA patients in China. It may reflect a rough estimate of the possible MMA-to-PA ratio in China, with a higher prevalence of MMA than other countries, in accordance with a previous report that the morbidity of PA is 1:150,000–1:120,000 whereas MMA reaches 1:65,000–1:5000 [26].

This study also found that the median ages of hospitalized patients with MMA or PA were both one-year-old. The reason for infant predominance is probably that organic acidurias can be diagnosed early owing to the development of neonatal mass screening nowadays. After MMA and PA are diagnosed correctly and treatment regimen are made, maintenance of protein restriction and drug treatment including L-carnitine, antimicrobial therapy and biotin supplementation are always implemented in outpatient or at home [1, 27, 28]. Moreover, organ transplantation can be even considered for severe cases in the early stage [5, 6, 29–35]. Regular monitoring of metabolic parameters and adjustment of therapeutic regimen are often carried out for transplant recipients during outpatient follow-up, which can effectively prevent episodes of metabolic decompensation, whilst decreasing hospital admissions thereafter for patients whose disease are well controlled. It probably also attributed to generally recognized triggers of acute decompensation (such as infection, fever, prolonged fasting, psychological stress, acute trauma, etc.), which have been increasingly familiar to patients or their parents, thus can be effectively avoided. In terms of MMA, the number of male pediatric patients was nearly equal to that of female, while the number of male PA patients was more than twice of female patients. This might indicate the sex gap in the incidence of the two diseases. Further population-based study is needed for this aspect.

In recent years, MMA and PA have been reported to be associated with increasing burden deserving intense attention [1, 3]. However, the financial cost has not yet been evaluated or quantified. In this study, the results showed that the median of total payments per hospitalization were relatively high, with more than RMB 4000 (about USD 580) for both diseases. Moreover, expenses for transportation and accommodation arising from a high proportion of cross-district hospitalization make things worse. We speculated that this has placed a certain amount of families under heavy financial burden.

Regarding to the distribution of hospital level, the study found that most of MMA and PA pediatric patients (80.96 and 76.21% respectively) were hospitalized in the highest level of hospitals, namely, tertiary class A hospital. Moreover, the hospitals located in Beijing, Chongqing, Shanghai and Jiangsu, admitted a majority of MMA or PA pediatric patients from other districts. This may be related to the unbalanced distribution of medical resources including experienced, specialized pediatricians and advanced equipment. For instance, measurement of serum/urine organic acid and genetic analysis has been generally available only in tertiary hospitals and rarely available in primary health care settings in China. These two diseases also require multidisciplinary team for nutritional, biochemical, neurodevelopmental and psychological assessment. Regular monitoring of metabolic parameters, developmental delay, long-term complications, compliance with therapy, along with overall nutritional status are strongly advised [1]. In China, another barrier for universal access to treatment for MMA and PA is the urban-rural inequity in health care. For example, most of the experts in inherited metabolic disease and liver transplant centres work in cities, especially big cities. Therefore, the medical level is unbalanced across different districts of China so far. Regardless of unbalanced distribution of medical resources across China, pediatric patients with MMA and PA generally have a favourable outcome when discharged, with only 1.69–2.23% in-hospital death as demonstrated by our study, far below the death rate of another hospital-based study in a single centre in Syria [17]. Our study provided the present distribution of health resources pertaining to MMA and PA in China, which possibly will contribute to advocating proper decentralization of health resources.

It has been already known that symptoms of MMA/PA may vary considerably and are nonspecific especially in childhood since multiple organ systems are affected [1, 3, 15, 29, 31, 36]. In our study, we found that patients are more likely to manifest with nervous system-related symptoms. And for laboratory findings, metabolic acidosis or anemia are the most common symptoms during hospitalization. Furthermore, this study, for the first time, provided information about the relative frequencies of signs of MMA/PA during hospitalization based on a large sample, although patients might be hospitalized due to other diseases unrelated to MMA/PA and some specific diagnosis might be neglected. Since most of patients with MMA/PA are under the care of primary physicians or specialists other than pediatrician experts in metabolic disorder, recognizing and identifying the signs in time during hospitalization is critical.

There are several limitations to our study. Firstly, the HQMS database is not able to distinguish each patient with repeated admissions, since ID numbers for children on the front page of medical records are usually unavailable. Secondly, we relied merely on ICD-10 codes for case identification, since we had no access to all medical records of studied population. This could have led to a less precise estimation of burden of hospitalized pediatric patients with MMA and PA to some extent. Thirdly, there were lack of follow-up outcomes for these pediatric patients in this study, as the study was based on sequential cross-sectional data.

Despite these limitations, our study provides epidemiological and clinical information of MMA and PA hospitalizations based on a nation-wide database, which would help us better understand the general profile of these two rare diseases in China.

## Conclusions

In conclusion, this study is the first one to provide epidemiological, health economic and clinical presentation information on hospitalized pediatric patients with MMA and PA in China based on a national database. An increasing hospitalization with various presentations and a heavy financial burden per hospitalization were observed, while the medial resources were still relatively centralized in only several districts, such as Beijing, Chongqing, Shanghai and etc.

## Data Availability

The datasets generated and/or analyzed during the current study are available from the corresponding author on reasonable request.
